# Organoid-based evaluation of SA55 and Pemivibart against evolving SARS-CoV-2 variants

**DOI:** 10.1080/22221751.2026.2713323

**Published:** 2026-08-03

**Authors:** Zhixin Wan, Ying Zhou, Fanchong Jian, Jiali Wu, Wei Xue, Man Chun Chiu, Yifei Yu, Qiaoshuai Lan, Shuxin Zhang, Zijun Zhao, Xiaoxin Zhu, Jingjing Huang, Yidong Yang, Yuhong Liu, Xinjie Meng, Lin Huang, Xiang Gao, Hin Chu, Cun Li, Yunlong Cao, Jie Zhou

**Affiliations:** a Department of Microbiology, School of Clinical Medicine, Li Ka Shing Faculty of Medicine, The University of Hong Kong, Hong Kong Special Administrative Region, People’s Republic of China; b Centre for Virology, Vaccinology and Therapeutics, Hong Kong Science and Technology Park, Hong Kong Special Administrative Region, People’s Republic of China; c Biomedical Pioneering Innovation Center, School of Life Sciences, Peking University, Beijing, People’s Republic of China; d BiomOrgan Ltd, Hong Kong, People’s Republic of China; e Laboratory of Advanced Biotechnology, Beijing Institute of Biotechnology, Beijing, People’s Republic of China; f State Key Laboratory of Emerging Infectious Diseases, Li Ka Shing Faculty of Medicine, The University of Hong Kong, Hong Kong Special Administrative Region, People’s Republic of China; g Pandemic Research Alliance Unit at The University of Hong Kong, The University of Hong Kong, Hong Kong Special Administrative Region, People’s Republic of China

**Keywords:** SARS-CoV-2, organoid, neutralization assasys, SA55, Pemivibart

## Abstract

Pemivibart, a class 1/4 monoclonal antibody (mAb), is currently the only FDA-authorized SARS-CoV-2 mAb under an Emergency Use Authorization (EUA) in clinical use. The emergence of subvariants, including KP.3.1.1 and XFG, raises concerns about antibody efficacy. SA55, a fully human class 1/4 mAb, is currently under clinical trial, including a nasal spray formulation. Using the well-validated organoid-based neutralization assays, we compared the potency and breadth of Pemivibart and SA55. Our results demonstrated a significant decrease in Pemivibart’s activity against KP.3.1.1 and XFG, with an approximately 80-fold increase in IC50 relative to the ancestral strain. Given KP.3.1.1’s strong reliance on the TMPRSS2 pathway for cell entry, we further demonstrated that combinational treatment with Pemivibart and the broad-spectrum S2 antibody results in potent neutralization. Notably, SA55 maintained potent neutralization (IC50 ≤ 40 ng/mL) across all tested variants. Topical administration, which models nasal spray, dramatically suppressed viral replication of BA.5.2 and XFG in organoid models. Our findings evidence the high potency of SA55, supporting its potential for clinical application against emerging SARS-CoV-2 variants.

Pemivibart (Pemgarda™/VYD222), an ADG-2-derived monoclonal antibody targeting conserved class 1/4 epitopes in the SARS-CoV-2 receptor-binding domain (RBD) [1], received FDA Emergency Use Authorization (EUA) in March 2024 for COVID-19 pre-exposure prophylaxis in immunocompromised individuals. As the only currently authorized monoclonal antibody in clinical use, Pemivibart has demonstrated broad neutralization against earlier Omicron lineages, including JN.1 [1]. However, the continuous evolution of SARS-CoV-2, particularly within JN.1-derived sublineages such as KP.3.1.1 and XFG, has raised concerns about potential erosion of antibody efficacy. XFG is a recombinant variant derived from the JN.1 sublineages LF.7.2.1 and LP.8.1.2. Compared to KP.3.1.1, XFG carries additional spike mutations including H445R, N487D, and T572I [2]. Recent studies reported conflicting neutralization activity against KP.3.1.1 carrying S31 deletion in the N-terminal domain, and RBD Q493E mutation. While several studies reported a significant (∼30–800-fold) reduction against KP.3.1.1 and related subvariants [3,4], data from the manufacturer, Invivyd, suggested its continued efficacy [5]. SA55, also a class 1/4 monoclonal antibody, was identified from a large library containing 13,000 antibodies isolated from SARS-CoV-2-vaccinated SARS convalescents using high-throughput single-cell sequencing and yeast display technology [6]. It has shown potent, broad neutralization across all variants of concern *in vitro* and *in vivo* [7]. Upon validation of its safety profile, SA55 has entered clinical trials for the treatment of COVID-19 patients, including a widely used nasal spray formulation in China [8].

We have established a robust human respiratory organoid culture system [9]. All differentiated respiratory organoid models, including nasal, airway, and alveolar organoids, faithfully simulate the multicellular composition and physiological functions of the native respiratory epithelium. These robust respiratory organoid models have demonstrated great potential for studying respiratory viruses and virus-host interactions [10]. Recently, we established and validated organoid-based neutralization assays for evaluating the potency of SARS-CoV-2 monoclonal antibodies. Through in-parallel comparison with conventional cell-line-based neutralization assays, we demonstrated that neutralization assays performed in nasal organoids adequately recapitulate the real-world efficacy of SARS-CoV-2 monoclonal antibody VIR-7831. In contrast, cell-line-based neutralization assays under-rated its potency, leading to the withdrawal from clinical use [11]. Here, we applied these well-validated organoid-based neutralization assays to compare the neutralization potency of Pemivibart and SA55 against SARS-CoV-2 variants, including the ancestral strain WT, as well as BA.4/5, EG.5.1, KP.3.1.1, and XFG.

Pseudovirus neutralization assay in nasal organoids revealed that Pemivibart effectively neutralized WT, BA.4/5, and EG.5.1 with 50% inhibitory concentration (IC50) values of 41, 194, and 819 ng/mL, respectively. However, its potency against KP.3.1.1 and XFG dropped markedly, with IC50 values of 3303 ng/mL for KP.3.1.1 and 2461 ng/mL for XFG, representing approximately 80-fold and 60-fold reductions compared to WT, respectively (Figure 1(A and B)). In-parallel neutralization assays in Huh-7, Vero E6-TMPRSS2, and 293T-ACE2 cell lines showed a consistent trend. Moreover, except for SARS-CoV-2 WT, Pemivibart rarely achieved more than 90% inhibition against the tested variants in these cell lines (Figure 1(A)). We further performed a live-virus neutralization assay in nasal organoids and verified that Pemivibart exhibited reduced activity against KP.3.1.1 and XFG (Figure 1(C)). Yet, the serum trough concentration of Pemivibart is 188 μg/mL at day 90 after a single-dose infusion of 4500 mg [12]. Given a serum-to-lung penetration rate of 6.5–12% [13], the antibody concentration at the respiratory mucosa is expected to reach 12–22 µg/mL. In nasal organoids, Pemivibart achieved >90% neutralization against live KP.3.1.1 and XFG at 10 µg/mL. Thus, the antibody may maintain its efficacy against these variants, despite the markedly reduced potency.

**Figure 1.**
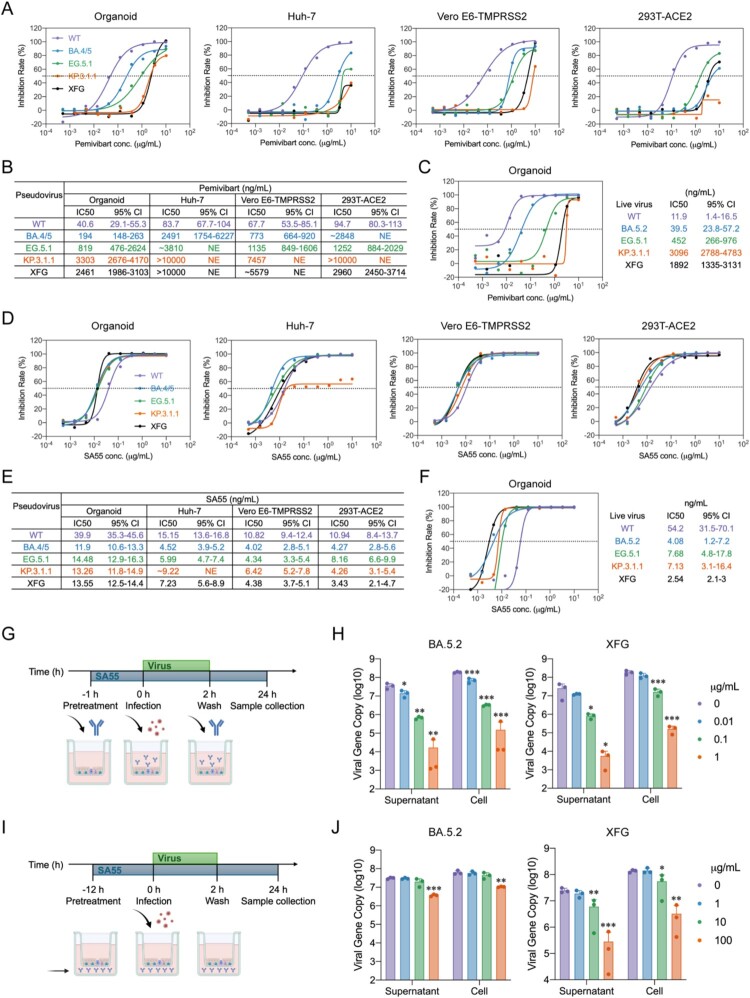
**SARS-CoV-2 neutralization by Pemivibart and SA55, and SA55 prophylaxis in human nasal organoids.** (A, B) Neutralization activity of Pemivibart against pseudoviruses. (A) Neutralization curves and (B) IC50 values against indicated SARS-CoV-2 pseudoviruses in human nasal organoids, Huh-7, Vero E6-TMPRSS2, and 293T-ACE2 cells. NE, not estimable due to incomplete neutralization plateau. (C) Neutralization activity of Pemivibart against authentic SARS-CoV-2 variants in nasal organoids (dashed line, 50% neutralization). (D, E) Neutralization efficacy of SA55 against pseudoviruses. (D) Neutralization curves and (E) IC50 values in nasal organoids and cell lines. (F) Neutralization efficacy of SA55 against authentic SARS-CoV-2 variants in nasal organoids. All neutralization data represent the mean of four technical replicates from a representative experiment. (G) Schematic of the experimental design for topical prophylaxis using SA55 IgG. Differentiated nasal organoids were pretreated apically with SA55 for 1 h before infection with SARS-CoV-2 variants. Viral RNA in media from top chambers and cell lysates was quantified at 24 hpi. (H) Efficacy of apical SA55 IgG treatment. Viral loads, measured by RT-qPCR, in apical supernatants and cell lysates of organoids infected with BA.5.2 (left) or XFG (right) following pretreatment with the indicated concentrations of SA55 IgG. (I) Schematic for basolateral exposure using SA55 dIgA. Organoids were pretreated basolaterally with SA55 dIgA for 12 h before infection. (J) Efficacy of basolateral SA55 dIgA treatment. Viral loads in apical supernatants and cell lysates from organoids infected with BA.5.2 (left) or XFG (right) after basolateral pretreatment with SA55 dIgA or control. Data are mean ± SD from three technical replicates of a representative experiment. *P ≤ 0.05, **P ≤ 0.01, ***P ≤ 0.001 by one-way ANOVA with Tukey’s multiple comparison test. All schematics were created with BioRender.com. CVVT, C. (2026).

SA55 potently neutralized all tested variants in pseudovirus assays performed in the organoids and cell lines (Figure 1(D and E)). Except for the compromised inhibition in Huh-7, SA55 achieved 100% inhibition with IC50 values below 40 ng/mL. The organoid-based live virus neutralization assay verified its high potency, with IC50 values of 7.13 ng/mL for KP.3.1.1 and 2.54 ng/mL for XFG (Figure 1(F)). Intriguingly, SA55 failed to neutralize the KP.3.1.1 pseudovirus in Huh-7 cells; the lowest neutralization against KP.3.1.1 pseudovirus was also observed in Pemivibart. Nonetheless, variable readouts of neutralization in different cell lines necessitate the application of physiologically relevant models, such as nasal organoids, for measuring antibody efficacy. Here, we demonstrate that SA55 substantially outperforms Pemivibart to neutralize SARS-CoV-2 emerging variants. SA55 and Pemivibart bind to different epitopes, although both are class 1/4 RBD antibodies that competitively block ACE2 engagement. Pemivibart targets residues including Q493 and F456, which are mutated in KP.3.1.1 (i.e. Q493E, F456L), leading to the reduced activity [3]. However, SA55 binds to a highly conserved region covering D405, R408, V503, and G504, thus bypassing the highly mutated hot spots and maintaining high neutralization potency and breadth [6].

We then modelled pre-exposure prophylaxis of SA55 via nasal spray and basolateral exposure in nasal organoids. Nasal organoid monolayers were treated with the IgG form of SA55 from the apical surface one hour before virus inoculation, which simulates prophylactic administration via nasal spray (Figure 1(G)). At a concentration of 0.1 µg/mL or higher, SA55 dramatically reduced the viral load of BA.5.2 and XFG by over 3 log units in the culture medium (Figure 1(H)). A similar level of viral reduction was observed intracellularly. Treatment with SA55 in a dimeric IgA (dIgA) form in the basolateral side of organoid monolayers also reduced viral replication of both variants (Figure 1(I and J)). However, a high concentration of 10 μg/mL or greater was required to achieve a significant viral reduction (Figure 1(J)), with a more pronounced inhibition observed against XFG compared to BA.5.2. Thus, topical administration of SA55 IgG in nasal organoids resulted in greater inhibition compared to basolateral treatment with SA55 dIgA, suggesting that local delivery to the mucosal surface may be more effective. Nonetheless, the efficacy of SA55 needs to be validated in clinical trials.

The less effective inhibition observed during basolateral treatment, intended to model systemic administration, may be related to the organoid model. Single-cell RNA sequencing analysis of nasal organoids shows high expression of polymeric immunoglobulin receptor (PIGR, data not shown), a key mediator of dIgA transcytosis. Additionally, a recent study also reported inefficient IgA transcytosis across respiratory epithelial cells, even with high PIGR expression [14]. Nonetheless, the current nasal organoids may require further improvement to accurately emulate *in vivo* dIgA transcytosis. On the other hand, the functional integrity and stability of the recombinant SA55 dIgA may need additional biochemical and functional verification and further optimization.

The reduced potency of Pemivibart against emerging variants prompted us to enhance its effectiveness via combinational therapy with broad-spectrum S2 antibodies. SARS-CoV-2 viruses primarily utilize Transmembrane Protease Serine 2 (TMPRSS2)-mediated surface entry in primary airway epithelial cells [15] and respiratory organoids [10], in which the *in vivo* dependency on this pathway was reproduced. S2-directed antibodies, unlike RBD-targeting antibodies, demonstrate broader spectrum activity and greater resilience against viral immune evasion. Based on our previous study, nasal organoids, with high TMPRSS2 expression and utilization of TMPRSS2-mediated surface membrane entry, uniquely revealed S2 antibodies’ *in vivo* protection [11]. Therefore, we explored the possibility of enhanced neutralization via combinational treatment with Pemivibart and S2 antibodies in nasal organoids.

First, we examined KP.3.1.1’s dependence on TMPRSS2-mediated entry in nasal organoids by measuring pseudovirus entry of WT, BA.4.5, and KP.3.1.1 in the presence of a TMPRSS2 inhibitor Camostat, or a cathepsin L/B inhibitor E64D (Figure S1(A)). Camostat effectively inhibited WT pseudovirus entry, while BA.4/5 showed reduced TMPRSS2 dependence, consistent with previous findings [10]. Notably, KP.3.1.1 pseudovirus entry restored the high sensitivity to Camostat, with a level comparable to WT pseudovirus entry, indicating its reliance on TMPRSS2-mediated entry in nasal organoids. Then, we evaluated neutralization of mono- and double-treatment with Pemivibart and one of two fusion peptide-targeting S2 antibodies, COV44-62 and COV44-79, against KP.3.1.1 pseudovirus entry. Double treatment with Pemivibart and either of the S2 antibodies demonstrated significantly enhanced neutralization of KP.3.1.1 pseudovirus entry than Pemivibart mono-treatment (Figure S1(B)). Here, two antibodies for double treatment were administered at 50% of the monotherapy concentration, ensuring the overall concentration was equal in both treatments. To validate the effect of double treatment, we performed live virus neutralization assays in nasal organoids. Double treatment with Pemivibart and S2 antibodies significantly potentiated the inhibition of KP.3.1.1 growth (Figure S1(C)), validating the additive effect of S2 antibodies to Pemivibart’s neutralization potency.

In summary, the organoid-based neutralization assays revealed Pemivibart’s reduced neutralization potency against the prevailing subvariants KP.3.1.1 and XFG, relative to prior variants [3,5]. A combinational treatment with S2-targeting antibodies elevated its activity against KP.3.1.1. Notably, SA55 demonstrates broader and more potent neutralization in nasal organoids and most cell lines. The IgG form of SA55 dramatically suppresses the viral growth of BA.5.2 and XFG upon topical administration. The high potency of SA55, especially its extraordinary resistance to SARS-CoV-2 mutations and evolution, is rooted in a rigorous and rational antibody discovery strategy [6]. Our study underscores the utility of respiratory organoids as a robust platform for evaluating and predicting the clinical effectiveness of neutralizing antibodies.

## Supplementary Material

Supplementary_Materials-clean.docx
