# Supplementary material for: Organoid-based evaluation of SA55 and Pemivibart against evolving SARS-CoV-2 variants
Source: Emerg Microbes Infect. 2026 Aug 3;15(1):2713323. doi: 10.1080/22221751.2026.2713323 (PMC13470914; doi:10.1080/22221751.2026.2713323)
Supplement: Supplementary file 1 [file TEMI_A_2713323_SM6453.docx]

**Supplementary Materials**

**Materials and Methods**

Table 1. Reagents and tools.

| Reagent or resource | Source | Identifier |
| --- | --- | --- |
| Antibodies |  |  |
| Pemivibart | MCE | Cat. #: HY-P990756 |
| SA55 | In-house | N/A |
| COV44-62 | In-house | N/A |
| COV44-79 | In-house | N/A |
| Inhibitors |  |  |
| Camostat | Sigma-Aldrich | Cat. #: SML0057 |
| E64D | Sigma-Aldrich | Cat. #: E8640 |
| Virus Strains | | |
| SARS-CoV-2 wild type | This paper | GenBank accession number: MT230904 |
| BA.5.2 | This paper | GISAID accession no. EPI_ISL_13777658 |
| EG.5.1 | This paper | GISAID accession no. EPI_ISL_18461518 |
| KP.3.1.1 | This paper | GISAID accession no. EPI_ISL_19534723 |
| XFG | This paper | GISAID accession no. EPI_ISL_20376607 |
| Experimental models | | |
| Human nasal organoids | This paper | Protocol UW21-695 |
| Vero E6-TMPRSS2 | JCRB cell bank | JCRB1819 |
| Huh-7 | JCRB cell bank | JCRB0403 |
| 293T-ACE2 | GeneCopeia | Cat. #: SL221 |
| Critical commercial assays | | |
| Bright-Glo™ Luciferase Assay System | Promega | Cat. #: E2620 |
| Software |  |  |
| GraphPad Prism 10 | N/A | N/A |

*Nasal organoid derivation and culture*

Nasal epithelial cells were collected noninvasively from healthy donors with approval from the Institutional Review Board of the University of Hong Kong/Hospital Authority Hong Kong West Cluster (Protocol UW21-695). Nasal organoids were established using respiratory organoid expansion medium (BiomOrgan) and maintained through serial passaging every 10-14 days for up to six months in the same medium. For differentiation, organoids were dissociated and seeded onto transwell inserts to generate polarized epithelial monolayers using differentiation medium (BiomOrgan), following established protocols [1-4]. All experiments were conducted using these differentiated nasal organoid monolayers cultured in 96-well or 24-well transwell formats.

*Cell line culture and viral propagation*

All cell lines were cultured at 37°C in an incubator with 5% CO_2_. Vero E6-TMPRSS2 cells were maintained in DMEM (Gibco) supplemented with 10% FBS (Gibco), 1% penicillin-streptomycin (Gibco), and 1 mg/mL G418 (Gibco). Huh-7 and 293T-ACE2 cells were cultured in DMEM containing 10% FBS and 1% penicillin-streptomycin, with 293T-ACE2 medium additionally supplemented with 100 μg/mL Hygromycin B. Cells were routinely passaged at 90% confluence using EDTA-trypsin (Gibco) at a 1:6 split ratio and underwent regular mycoplasma testing to ensure contamination-free cultures.

SARS-CoV-2 isolate HKU-001a (wild type, GenBank accession number MT230904), the Omicron subvariant BA.5.2 (GISAID accession no. EPI_ISL_13777658), EG.5.1 (GISAID accession no. EPI_ISL_18461518), KP.3.1.1 (GISAID accession no. EPI_ISL_19534723), and XFG (GISAID accession no. EPI_ISL_20376607) were plaque-purified and amplified in Vero E6-TMPRSS2 cells. Viral titers were determined by standard plaque assay as previously described.

*Production of SARS-CoV-2 pseudovirus*

Lenti-X 293T cells (Takara, 632180) were cultured in DMEM supplemented with 10% FBS and 1% P/S. SARS-CoV-2 variant pseudoviruses were produced by co-transfecting an HIV backbone reporter plasmid (pNL4-3-R-E-luciferase) and a pcDNA3.1 vector (Invitrogen) encoding the respective spike proteins into Lenti-X 293T cells (Takara Bio). After 48-72 hours of incubation, supernatants were harvested, filtered through a 0.45 μm membrane, and centrifuged at 800 *g* for 5 minutes to remove cell debris. Viral titers were quantified by detecting luciferase activity using the Bright-Glo Luciferase Assay System (Promega). Controls were transfected with equal amounts of empty vector instead of the spike protein-expressing plasmid.

*Neutralization assays*

Neutralization assays were performed by incubating pseudoviruses with serially diluted mAbs at 37°C for one hour. Subsequently, the virus-antibody mixtures were added to Huh-7 cells, Vero E6-TMPRSS2 cells, and 293T-ACE2 cells seeded in 96-well plates, as well as nasal organoids grown in 96-well transwell plates. The cells and organoids were incubated for 72 hours before being subjected to luciferase assays. Each assay plate included two controls, a virus control (cells incubated with pseudoviruses only) and a background control (cells or organoids without pseudovirus exposure). A decrease in luminescence, measured as relative luminescence units (RLUs), in the presence of mAbs indicated neutralization activity. The IC50 was defined as the antibody dilutions that resulted in a 50% reduction in RLUs compared to the virus control after subtracting background luminescence. IC50 values were calculated using a four-parameter nonlinear regression model in GraphPad Prism 10 (GraphPad Software).

The live virus neutralization assay was conducted within a certified Biosafety Level 3 (BSL-3) laboratory. SARS-CoV-2 variants were incubated at a multiplicity of infection (MOI) of 0.01 to 0.1 with serially diluted mAbs in 96-well plates for one hour at 37°C. The mixtures were then transferred onto organoid monolayers cultured in 96-well transwell plates and incubated for 2 hours at 37°C. After washing, the infected organoids were maintained in basal medium in both the top and bottom chambers. At 24 hpi, 50 μL culture medium was collected from the top chamber of each well, mixed with AVL lysis buffer, and subjected to RNA extraction using the RNeasy Pure mRNA Bead Kit (QIAGEN). Viral loads were quantified by RT-qPCR as described previously [3]. The neutralization efficacy was determined by calculating the percentage reduction in viral load in antibody-treated organoids relative to untreated controls. IC50 values were derived using four-parameter nonlinear regression analysis in GraphPad Prism 10.

*Modeling prophylaxis in nasal organoids*

To model nasal spray administration, we incubated nasal organoid monolayers apically with indicated concentrations of SA55 IgG for 1 hour. The monolayers were then infected apically with the indicated viruses at an MOI of 0.01 for 2 hours at 37°C. After infection, the organoids were washed twice and then incubated with SA55 IgG in the apical chamber. At 24 hpi, culture medium in the upper chamber and cell lysates were collected for viral load quantification by RT-qPCR.

For basolateral administration, we incubated nasal organoid monolayers with indicated concentrations of SA55 dIgA in the bottom chamber for 12 hours. The monolayers were subsequently infected apically with the indicated viruses at an MOI of 0.01 for 2 hours at 37°C. Following infection and two washes, the monolayers were incubated with SA55 dIgA in the bottom chamber. At 24 hpi, culture medium in the upper chamber and cell lysates were collected for viral load quantification by RT-qPCR.

*Inhibition of pseudovirus entry*

Nasal organoids were pretreated with 50 µM Camostat (Sigma-Aldrich), 50 µM E64D (Sigma-Aldrich), or DMSO control for 1 hour at 37°C. They were then spin-inoculated with SARS-CoV-2 WT, BA.4/5, and KP.3.1.1 pseudovirions in the presence of the respective inhibitor by centrifugation at 300 × *g* for 1 hour. After inoculation, organoids were washed twice and lysed 72 hours later to measure luciferase activity.

*Source of monoclonal antibodies*

For this study, the mAb Pemivibart was commercially sourced (HY-P990756), whereas SA55 was obtained from established in-house collections as described in our previous publication [5]. Prof Zheng Zhang kindly provided COV44-62 and COV44-79, which have been described previously [4].

*Statistical analysis*

Statistical analysis was conducted using GraphPad Prism 10.0. Student's t-test or ANOVA was used to assess statistical significance, as specified in the figure legends. **P* ≤ 0.05, ***P* ≤ 0.01, ****P*≤ 0.001.

**SM Figure 1**

**SM Figure 1. Enhanced neutralization of KP.3.1.1 by Pemivibart and S2 antibody combination in nasal organoids.** (A) Pseudovirus entry into nasal organoids was assessed in the presence or absence of serine protease inhibitors using a luciferase assay. Results are expressed as relative luciferase activity in inhibitor-treated samples compared to DMSO-treated controls. Data are mean ± SD from six technical replicates of a representative experiment. Statistical significance was determined by one-way ANOVA with Tukey’s multiple comparisons test. ****P* ≤ 0.001; ns, not significant. (B) Neutralization activity of Pemivibart and COV44-62 (left) or COV44-79 (right) combination against KP.3.1.1 pseudovirus in nasal organoids. (C) Neutralization efficacy of Pemivibart and COV44-62 (left) or COV44-79 (right) combination against authentic KP.3.1.1 virus in nasal organoids. All neutralization data represent the mean of four technical replicates from a representative experiment.

**References**

1.	Zhou, J., et al., *Differentiated human airway organoids to assess infectivity of emerging influenza virus.* Proc Natl Acad Sci U S A, 2018. **115**(26): p. 6822-6827.

2.	Li, C., et al., *Human airway and nasal organoids reveal escalating replicative fitness of SARS-CoV-2 emerging variants.* Proc Natl Acad Sci U S A, 2023. **120**(17): p. e2300376120.

3.	Li, C., et al., *Human respiratory organoids sustained reproducible propagation of human rhinovirus C and elucidation of virus-host interaction.* Nat Commun, 2024. **15**(1): p. 10772.

4.	Wan, Z., et al., *Organoid-based neutralization assays reveal a distinctive profile of SARS-CoV-2 antibodies and recapitulate the real-world efficacy.* Proc Natl Acad Sci U S A, 2025. **122**(35): p. e2509616122.

5.	Cao, Y., et al., *Rational identification of potent and broad sarbecovirus-neutralizing antibody cocktails from SARS convalescents.* Cell Rep, 2022. **41**(12): p. 111845.
